# Evaluation of the methodology of independent Community Advisory Boards in health products research and development: a mixed-methods cross-sectional survey study

**DOI:** 10.1186/s40900-026-00866-9

**Published:** 2026-03-20

**Authors:** Rob Camp, Simona Borroni, Hilde DeKeyser, Mark Hees, Aleksandra Leijenhorst, Mohsharif Nasrulloeva, David Trigos, François Houÿez

**Affiliations:** 1 EUPATI Spain, Lugo, Spain; 2 Dravet Syndrome European Federation (DSEF), Dravet CAB, Paris, France; 3 Cystic Fibrosis Europe (CFE), Cystic Fibrosis CAB, Brussels, Belgium; 4 Myotonic Dystrophy CAB, Amsterdam, Netherlands; 5 Stichting Spierkracht, Limb-girdle Muscular Dystrophy (LGMD) CAB, Amsterdam, Netherlands; 6 European Multiple Sclerosis Platform (EMSP), Multiple Sclerosis CAB, Brussels, Belgium; 7 EURORDIS, Paris, France; 8 EUPATI Spain, Av. Alvaro Cunqueiro 27, 2D, Foz, Lugo, 27780 Spain

**Keywords:** Patient engagement in research, Patient engagement impact, Collaborative decision-making (regulatory and HTA), Drug research and development, Patient unmet needs, Rare diseases, Survey methods

## Abstract

**Background:**

EUPATI Spain and EURORDIS have implemented a patient engagement model in health product research and development (R&D): international, independent Community Advisory Boards (CABs), where patients collaborate with health product companies in patient-led CAB meetings. We have evaluated the methodology of these CAB meetings.

**Methods:**

We conducted a mixed-methods cross-sectional survey study from 2022 to 2023 to analyze the satisfaction level and perceived usefulness of CAB meetings between CAB members and company representatives across 15 CAB meetings. After each meeting, participants received an online survey comprising ten closed-ended questions on a five-point Likert scale and two open-ended questions about key takeaways and issues to improve. Written responses to open-ended questions were analyzed by AI-assisted ATLAS.ti, which categorized and quantified them semantically, and by contextual relevance and frequency. Characteristics of meetings and participants were reported, and survey responses were compared between CAB members and company representatives.

**Results:**

Most meetings were hybrid (53.3%). A total of 252 participants attended the meetings (158 [62.7%] CAB members, 94 [37.3%] company representatives). The overall survey response rate was 54.8%, 59.5% among CAB members and 46.8% among company representatives. Most participants found the meetings useful (86.0% CAB, 93.2% company), were satisfied with the topics addressed (77.4% CAB, 93.2% company), and met their expectations (76.7% CAB, 93.2% company). Both groups considered that the company understood the CAB’s point of view (80.2% CAB, 90.9% company). Still, only 59.3% of CAB members thought the company would reconsider its plans, vs. 88.6% of company representatives. The three main takeaway areas were “Collaboration and Partnerships”, “Study Design and Protocols”, and “Communication and Education”. The main issue to improve was “Meeting Organization and Structure”.

**Conclusions:**

Overall, the satisfaction level and perceived usefulness of the CAB meetings were favorable. CAB members exhibited less favorable responses to all closed-ended questions than company representatives, especially regarding the possible influence on company actions. The open-ended responses reflected higher levels of agreement and collaboration, supporting the CAB model’s potential. Some issues, mainly related to meeting organization and the hybrid format, need improvement. Future studies are required to assess the actual impact of CAB meetings on R&D.

**Plain English summary:**

EUPATI Spain and EURORDIS have implemented a patient engagement model in health product research and development: Community Advisory Boards (CABs). CABs are international, independent groups of patient representatives selected by their community who meet regularly with health product companies to discuss a wide range of topics, from study design to compassionate use of therapies. We have analyzed the satisfaction level and perceived usefulness of CAB meetings for CAB members and company representatives who attended 15 CAB meetings from 2022 to 2023, using a seven-minute online survey after each meeting. The survey included ten closed-ended questions and two open-ended questions on key takeaways and issues to improve. The feedback was highly positive. Most participants found the meetings useful, were satisfied with the topics addressed, and met their expectations. However, CAB members exhibited slightly less favorable responses to all closed-ended questions than company representatives, especially regarding the influence of CAB meetings on future company actions. The three main takeaway areas were “Collaboration and Partnerships”, “Study design and Protocols”, and “Communication and Education”; and the main issue to improve was “Meeting Organization and Structure”. Overall, the results were favorable. Although the closed-ended questions showed less confidence among CAB members on the future influence of the meetings, the comments from the open-ended questions reflected higher levels of agreement and collaboration and supported our CAB model’s potential. Some issues, mainly related to meeting organization and the hybrid format, need improvement. Future studies are required to assess the actual impact of CAB meetings on companies’ actions.

**Supplementary Information:**

The online version contains supplementary material available at 10.1186/s40900-026-00866-9.

## Background

Over the last three decades, the patient community, regulatory authorities, and funding bodies have redefined the patient’s role in research and development (R&D) of health products, shifting from mere study participants to a more patient-centered approach [1–3]. Patient engagement (PE) in R&D has been defined as “the active, meaningful, and collaborative interaction between patients and researchers across all stages of R&D, where decision-making is guided by patients’ contributions as partners, recognizing their experiences, values, and expertise” [4]. It improves scientific discussions and impacts on health product developers (hereafter referred to as ‘companies’) [5]. An array of PE initiatives in R&D has been launched, usually organized by healthcare providers, companies, and regulatory authorities [3, 6, 7]. The European Medicines Agency (EMA) has involved patients’ representatives in scientific committees, consultations, or advisory expert groups [8]. Likewise, the Food and Drug Administration (FDA) has developed, among other programs, the FDA-led Patient-Focused Drug Development (PFDD) and the externally-led PFDD meetings [9].

Despite the growing recognition of PE’s importance in health products R&D, patient leadership, even patient co-leadership, remains the exception rather than the rule [10–12]. Since patients’ and companies’ needs and objectives may differ [13, 14], the European Patients’ Academy on Therapeutic Innovation (EUPATI) Spain [15, 16] and the European Organization for Rare Diseases (EURORDIS) [17] have implemented an international patient-led approach to PE: Community Advisory Boards (CABs) within the EuroCAB program [18, 19].

CABs are international, independent groups, sometimes legal entities, sometimes a less formal collection of patient representatives (individual patients, carers, patient advocates, patient organization representatives, and patient experts) selected by their community, according to the requirements and procedures established by EUPATI Spain/EURORDIS (see Additional File 1) [19, 20]. They meet regularly with companies to discuss a wide range of topics, from study design to compassionate use of drugs. Unlike traditional PE approaches, CABs maintain independent governance structures and develop their own methodologies for measuring success [19, 20]. They operate under the five EUPATI Spain core principles [21]: 1) Patient-driven leadership, 2) Independent and autonomous management, 3) Open participation, 4) Transparent processes, and 5) Adherence to good engagement practices. Currently, there are 12 CABs at various stages of development (see Supplementary Table 1, Additional File 2).

Despite the wide range of PE initiatives in R&D, few studies have systematically evaluated the methodologies used and how the patient-company relationship is implemented [22–26]. Analyzing this is crucial for optimizing PE and aligning the needs and objectives of patients and companies [11]. We have evaluated the methodology of the EUPATI Spain/EURORDIS CAB model in health products R&D. To achieve this objective, we conducted a mixed-methods cross-sectional survey study from 2022 to 2023 that analyzed the differences in the satisfaction level and perceived usefulness of CAB meetings between CAB members and company representatives, across 15 CAB meetings covering both rare and non-rare diseases.

## Methods

### Settings and study design

This was a mixed-methods cross-sectional survey study conducted between November 2022 and December 2023. A total of 15 meetings were held, either face-to-face in Barcelona, Baltimore, Brussels, Madrid, and Prague, online, or as hybrid sessions. A completed GRIPP2 short form reporting checklist is given in Additional File 3.

## CAB meetings

Depending on the capacity/availability of the CABs, six CABs agreed to participate in the study covering both rare and non-rare diseases. These diseases were classified according to the definition of the European Parliament (1999) of rare diseases as those that are life-threatening, chronically debilitating, and of low prevalence (fewer than 5 per 10,000 people) [27, 28]. Most CABs were dedicated to one disease, whether a non-rare disease, such as multiple sclerosis (European Multiple Sclerosis Platform, EMSP), or a rare disease, such as atypical hemolytic uremic syndrome (the Global aHUS Partnership, GaP), cystic fibrosis (Cystic Fibrosis Europe, CFE), Dravet syndrome (Dravet Syndrome European Federation, DSEF), and limb-girdle muscular dystrophy (LGMD CAB). The Pulmonology CAB comprised a group of pulmonary diseases (asthma, bronchiectasis, chronic obstructive pulmonary disease [COPD], and cystic fibrosis [CF]), all non-rare diseases except for CF. For more information about the specific CABs, see Supplementary Table 1, Additional File 2.

EUPATI Spain coordinated meetings regarding the preparation, contract administration, logistics, and management of confidentiality disclosure agreements. Meeting modality could be face-to-face, online, or hybrid, with sessions lasting 3–7 hours (duration agreed in advance). Participants were invited to take part via the individual disease network. Participation was voluntary, and participants received financial support from EUPATI Spain. Flights, accommodation, and subsistence were covered, along with a nominal honorarium for each participant.

CAB members and company representatives collaboratively draft the agenda, allowing new ideas and considerations to emerge and develop during the meetings. Confidentiality agreements were in place, and minutes were recorded. After each meeting, the CAB drafted a letter outlining the next steps for the collaboration to be reviewed and further developed, or at the very least considered, with the company providing reasons for either proceeding with them or not.

The meeting findings were delivered to each company’s *community liaison*, who relayed them to their team to consider them in their future decision-making and actions, and the CABs used these results to plan their next steps.

After the end of each meeting, participants received a brief seven-minute online survey to provide feedback on the meeting’s satisfaction and usefulness, and to plan the next steps.

## Online survey

After each meeting, the EUPATI Spain coordinator sent the Google Docs link to the seven-minute online survey to all CAB members and company representatives (see Additional File 4, Online Survey). Participants received a reminder email, and the survey was available for approximately 10 days. Participation in the survey was voluntary and anonymous; only the stakeholder group (CAB member vs. company representative) was registered.

The survey combined quantitative methodology, using closed-ended questions, with qualitative methodology, using open-ended questions, to assess the satisfaction level and perceived usefulness of the meetings by the two groups of participants (CAB members/company representatives). It included ten closed-ended questions (Q), which evaluated the agreement level by a five-point Likert scale on five items: “Usefulness and Satisfaction” (Q1 and Q2); “CAB and Company preparation” (Q3 and Q4); “Trust, transparency, and openness” (Q5); “Expectations” (Q6); and “CAB-Company relationship” (Q7–Q10). It also included two open-ended questions about “Key meeting takeaways” (Q11) and “Issues to improve” (Q12).

EUPATI Spain formatted the questions based on the outcomes from a previous EuroCAB program case study within the Patients Active in Research And Dialogue for an Improved Generation of Medicines (PARADIGM) project [29], which analyzed a patient engagement monitoring and evaluation (PEME) framework for CAB meetings [19]. EUPATI Spain collaborated with the Free University of Amsterdam (Vrije Universiteit Amsterdam) to align the questions with the objectives of the CAB program.

We used an online survey to assess the satisfaction level and perceived usefulness because it is a widely employed, cost-effective, and easy-to-administer tool [30, 31]. In the context of CAB meetings with participants from different continents and varying availability, which poses a coordination challenge, we believe that other tools, such as focus groups, would be more difficult to implement and would result in lower participation. Surveys can also combine quantitative methods, using multiple-response closed-ended questions, with qualitative methods, through open-ended questions designed to learn patients’ opinions, experiences, or perceptions [12, 32]. This allows responses to be quantified while providing a space for responders to express their concerns freely.

## Meeting and participants’ characteristics

Meeting variables included the total number of meetings and the number of disease-specific CAB meetings (aHUS/CFE/Dravet/LGMD/MS/Pulmonology), as well as their modality (face-to-face/online/hybrid). Variables of participants who attended the meetings included stakeholder group (CAB member/company representative) and, for CAB members only, gender (man/woman/non-binary) and disease-specific CAB (aHUS/CFE/Dravet/LGMD/MS/Pulmonology). Gender and disease-specific CAB of the company representatives were not included for confidentiality reasons. For participants who answered the survey, only the stakeholder group (CAB member/company representative) was included as a variable.

## Data analysis

No formal sample size calculation or power analysis was performed. All participants who answered the survey were included by non-probability consecutive sampling, and all surveys that were fully or partially completed were included in the analysis, except for the MS CAB. Missing data for each question were indicated. The MS CAB independently designed its closed-ended questions to focus on its specific objectives, while sharing the same open-ended questions with the other CABs. To avoid bias, the closed-ended questions of the MS CAB were excluded from the analysis. Responses to the open-ended questions were included.

The characteristics of meetings and participants were summarized using descriptive statistics, with numerical and categorical variables reported by frequency (number) and percentage. Like categorical variables, the closed-ended questions were outlined by frequency (number) and percentage, and compared between CAB members and company representatives. The answers “Totally agree” and “Agree” were considered as “Agree”, and the answers “Totally disagree” and “Disagree” were considered as “Disagree”. The meeting and participants’ characteristics, as well as the closed-ended questions, were analyzed using Microsoft 365 Excel.

Open-ended responses were handled using the Artificial Intelligence (AI) sequencer ATLAS.ti to categorize the written responses and quantify the results (phrases to categories), a methodology commonly applied in qualitative opinion research [14, 33–38]. It uses an inductive thematic analysis approach without applying a pre-defined coding framework. Text segments from written responses were coded based on semantic meaning, then grouped into thematic categories based on frequency and contextual relevance. Once the categories were identified, each response was assigned to one of these categories. AI-assisted categorization was subsequently reviewed and refined by the investigators. Responses in each category were summarized and quantified as frequencies (numbers) and percentages. Responses that were particularly meaningful or effectively represented participants’ views were selected and presented in a table. Confidential comments were excluded from the analysis.

## Results

### Meeting characteristics

A total of 15 meetings were held, mainly for the aHUS CAB (5, 33.3%), the Dravet CAB (4, 26.6%), and the CFE CAB (3, 20.0%). Most were hybrid sessions (8, 53.3%). The results of the meeting characteristics are provided in Table 1.

**Table 1:** Meeting characteristics

Characteristic	n (%)
Total number of meetings	15 (100.0)
aHUS	5 (33.3)
Dravet	4 (26.6)
CFE	3 (20.0)
LGMD	1 (6.7)
MS	1 (6.7)
Pulmonology	1 (6.7)
Modality	
Hybrid	8 (53.3)
Online	5 (33.3)
Face-to-face	2 (13.4)

Abbreviations: aHUS, Global atypical Hemolytic-Uremic Syndrome Partnership; CFE, Cystic Fibrosis Europe; Dravet, Dravet Syndrome European Federation; LGMD, Limb-Girdle Muscular Dystrophy CAB; MS, European Multiple Sclerosis Platform; Pulmonology, a cross-section of pulmonary diseases including asthma, bronchiectasis, chronic obstructive pulmonary disease (COPD), and cystic fibrosis (CF)

## Participants’ characteristics

A total of 252 participants attended the meetings, 158 (62.7%) CAB members and 94 (37.3%) company representatives. Of the CAB members, the majority belonged to the aHUS CAB (70, 44.3%). The total response rate was 54.8% (138 of 252 participants): 59.5% (94 of 158) among CAB members and 46.8% (44 of 94) among company representatives. The results of the participants’ characteristics are provided in Table 2.

**Table 2:** Participants’ characteristics

Characteristic	n (%)
**Participants who attended the meeting**	252 (100.0)
**CAB members**	158 (62.7)
Gender	
Women	99 (62.6)
Men	59 (37.4)
Non-binary	0 (0.0)
CAB	
aHUS	70 (44.3)
CFE	39 (24.7)
Dravet	20 (12.6)
MS	11 (7.0)
Pulmonology	10 (6.3)
LGMD	8 (5.1)
**Company representatives**^**a**^	94 (37.3)
**Participants who answered the survey**^**b**^	138 (100.0)
Stakeholder group	
CAB member	94 (68.1)
Company representative	44 (31.9)

Abbreviations: aHUS, Global atypical Hemolytic Uremic Syndrome Partnership; CAB, Community Advisory Board; CFE, Cystic Fibrosis Europe; Dravet, Dravet Syndrome European Federation; LGMD, Limb-Girdle Muscular Dystrophy CAB; MS, European Multiple Sclerosis Platform; Pulmonology, a cross-section of pulmonary diseases including asthma, bronchiectasis, chronic obstructive pulmonary disease (COPD), and cystic fibrosis (CF)

A Information about the company representatives was not included for confidentiality reasons

B Members of the MS CAB had alternate closed-ended questions (Q1-10), but the same open-ended questions (Q11-12). Only the results of their open-ended questions (Q11-12) were included in the analysis

## Closed-ended questions

**Fig. 1:**
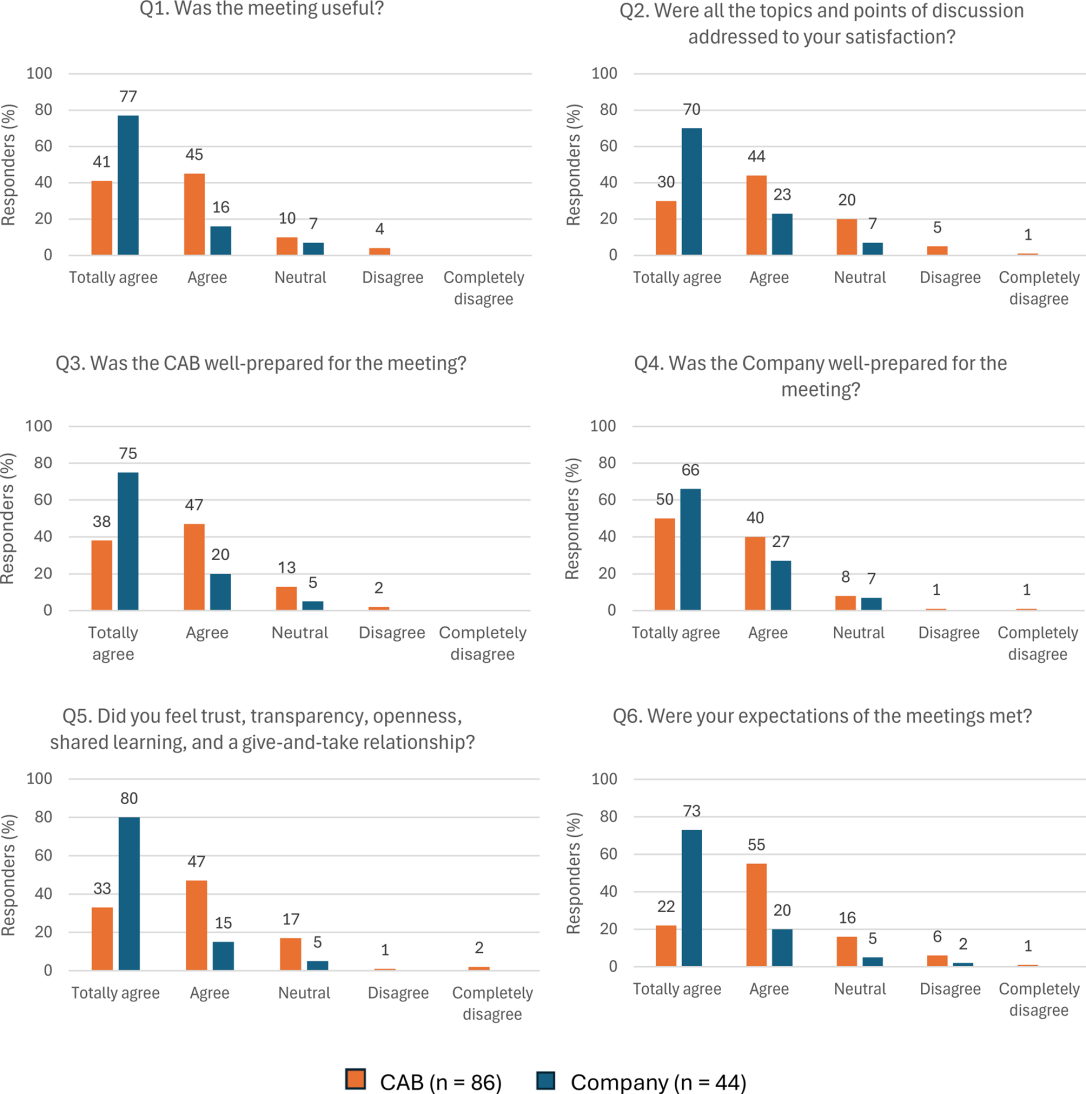
Answers to closed-ended questions Q1–Q6 by stakeholder group. Abbreviations: CAB, Community Advisory Board; Q, question

**Fig. 2:**
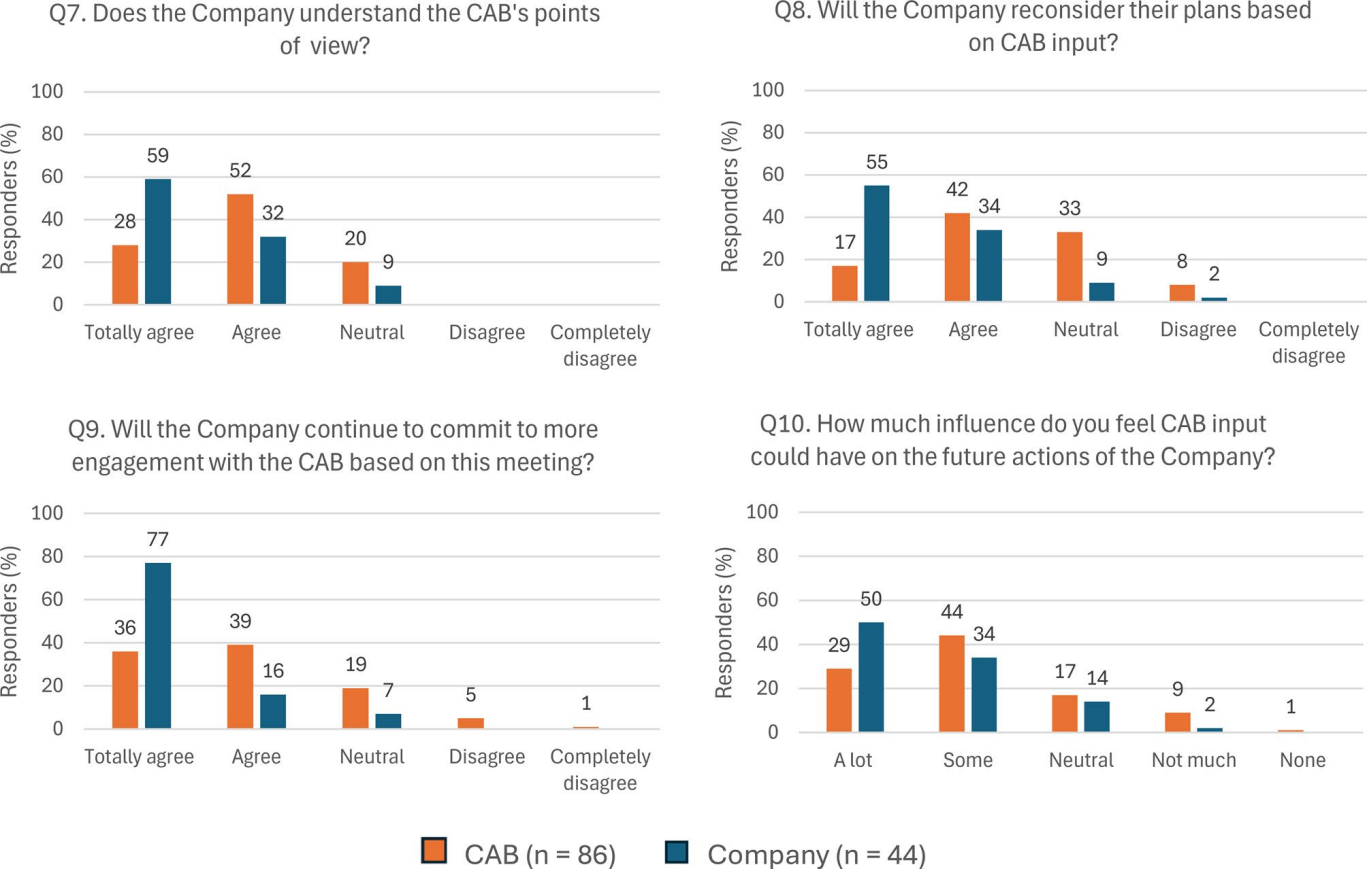
Answers to closed-ended questions Q7–Q10 by stakeholder group. Abbreviations: CAB, Community Advisory Board; Q, question

All participants who answered the survey completed the closed-ended questions (*n* = 130); 86 were CAB members, and 44 were company representatives (the 8 MS CAB members were excluded from the analysis). Fig. 1 summarizes Q1–Q6 answers, and Fig. 2 summarizes Q7–Q10 answers, by stakeholder group.

*Q1 and Q2,* “*Usefulness and Satisfaction”*: 74 CAB members (86.0%) and 41 company representatives (93.2%) agreed that the meeting was useful, and 64 CAB members (74.5%) and 41 company representatives (93.2%) agreed that all the topics of discussion were addressed to their satisfaction.

*Q3 and Q4,* “*CAB and Company preparation”*: 73 CAB members (84.8%) and 42 company representatives (95.4%) agreed that the CAB was well-prepared for the meeting, and 77 CAB members (89.5%) and 41 company representatives (93.2%) agreed that the company was well prepared for the meeting.

*Q5, “Trust, transparency, and openness”*: 68 CAB members (79.1%) and 42 company representatives (95.4%) agreed that they felt trust, transparency, openness, shared learning, and a give-and-take relationship.

*Q6, “Expectations”*: 66 CAB members (76.7%) and 41 company representatives (93.2%) stated that their expectations were met.

*Questions 7-10, “CAB-Company relationship”* 69 CAB members (80.2%) and 40 company representatives (90.9%) agreed that the company understood the CAB’s point of view; 51 CAB members (59.3%) and 39 company representatives (88.6%) agreed that the company would reconsider their plans based on CAB input; 65 CAB members (75.6%) and 41 company representatives (93.2%) agreed that the company would continue to commit to more engagement with the CAB based on the meeting; and 63 CAB members (73.2%) and 37 company representatives (84.0%) agreed that the CAB input would influence the future research/actions of the company.

## Open-ended questions

For the first open-ended question (Q11), “What were your three key takeaways from this CAB meeting?”, the response categories generated by ATLAS.ti AI and verified by the principal investigator were as follows: “Collaboration and Partnerships”, “Study Design and Protocols”, “Communication and Education”, “Access and Inequalities”, “Organization and Logistics”, “Mental Health”, “Health care”, and “Other” (including drug- or study-specific confidential comments, or those otherwise difficult to categorize).

**Fig. 3:**
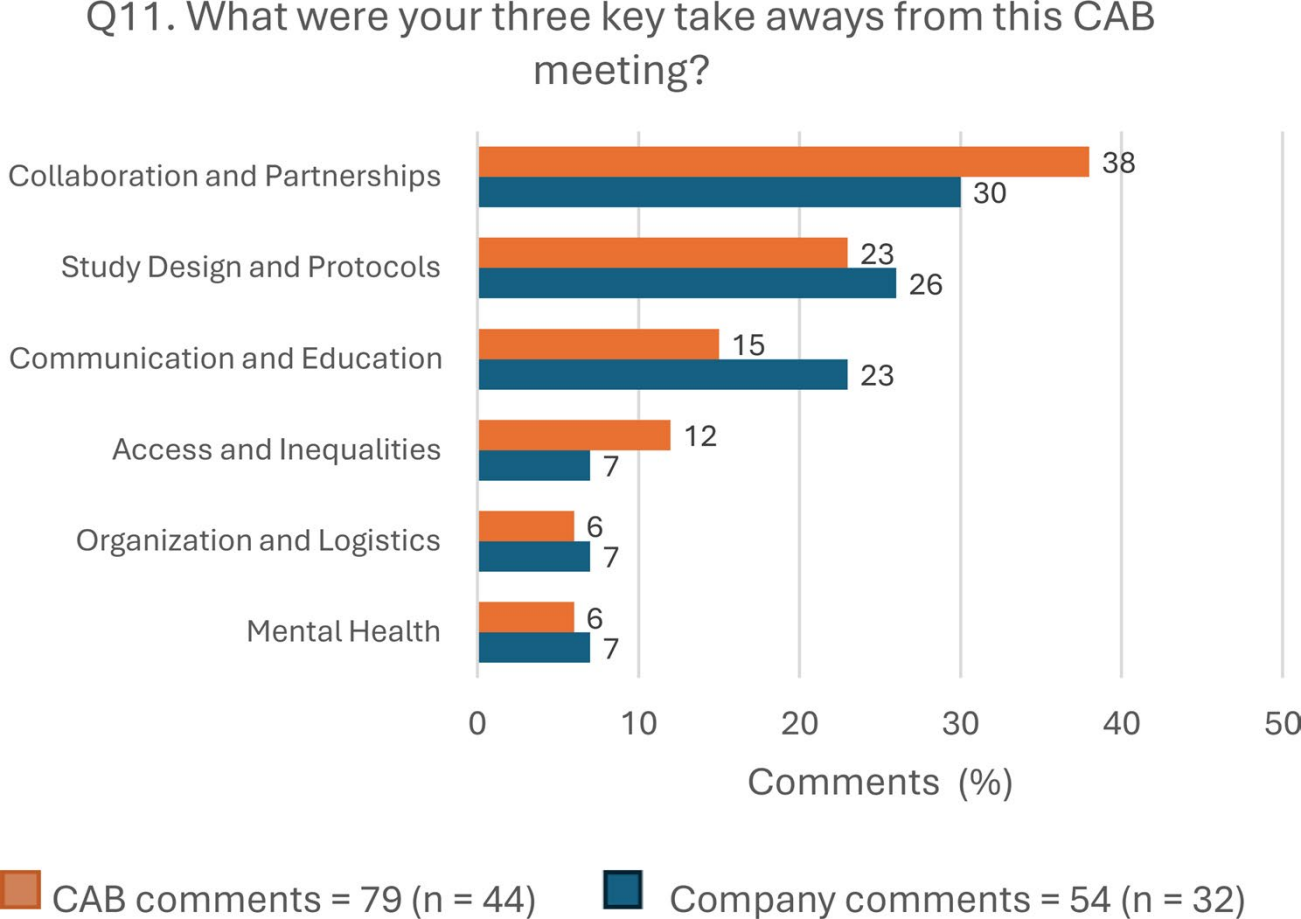
Answers to open-ended question 11 by stakeholder group. Abbreviations: CAB, Community Advisory Board; Q, question

A total of 44 CAB members answered this question, providing 79 comments, and 32 company representatives, providing 54 comments. Most comments for CAB members and company representatives were related to the same three takeaways: “Collaboration and Partnerships” (30, 38.0% CAB; 16, 29.6% company), “Study Design and Protocols” (18, 22.8% CAB; 14, 25.9% company), and “Communication and Education” (12, 15.2% CAB; 12, 22.2% company). Figure 3 shows the Q11 answers by stakeholder group, and Table 3 highlights the more representative comments.

**Table 3:** Comments to Q11: What were your three key takeaways from this CAB meeting?

Category	CAB	Company representatives
Collaboration and Partnerships	“There is a good, open atmosphere.” “They are open to patient input.” “The company saw the usefulness of the CAB and will want to continue the interaction.”	“Great to establish a strong working relationship where we can gain critical insights as we move forward.” “Long-term partnership”
Study Design and Protocols	“Specifics within various studies (type of therapy, mutations, new trials, coinfections)”	“Insight into Phase III study design” “Input on diary types” “Great suggestions for making planning more targeted (living, studying, aging, motherhood, diet)”
Communication and Education	“Education to doctors and patients regarding individualized treatment and discontinuing treatment” “Stigma”	“If CAB members don’t understand pathways, the rest of the (broader) community will likely need clarification.”
Access and Inequalities	“Global access, worldwide access vs. profit”	“Continuous need to update on access”
Organization and Logistics	“Good atmosphere” “The sponsor was well-prepared and listened actively.”	“The length of the agenda played out fine this time.”
Mental Health	“More research is needed on mental health side effects.”	“The openness about the mental health impacts on people living with the disease and their caregivers is appreciated.”

Abbreviations: CAB, Community Advisory Board; Q, question

For the second open-ended question (Q12), “Should we have done any part of the meeting differently?”, the response categories generated by ATLAS.ti AI were as follows: “Positive Comments” (those who essentially answered “No”), “Meeting Organization and Structure”, “Meeting Format and Audio-visual (AV)”, “Length of Meeting”, “Preparation and Logistics”, and “Facilitation and Moderation”.

**Fig. 4:**
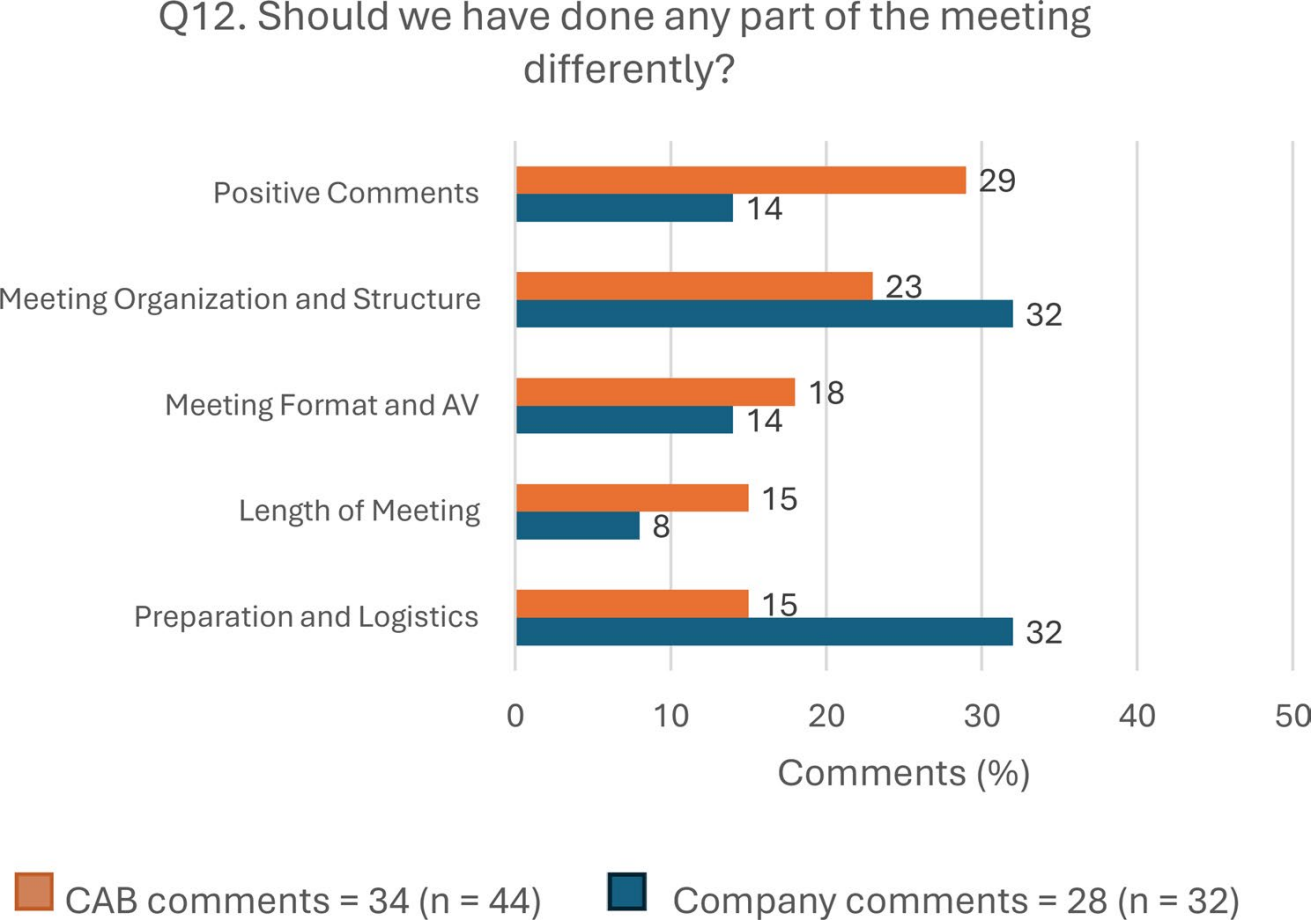
Answers to open-ended question 12 by stakeholder group. Abbreviations: AV, audio-visual; CAB, Community Advisory Board; Q, question

A total of 44 CAB members answered this question with 34 comments. Most were “Positive Comments” (10, 29.4%), and those about issues to improve were mainly related to “Meeting Organization and Structure” (8, 23.5%), “Meeting format and AV” (6, 17.6%), “Length of meeting” (5, 14.7%), and “Preparation and Logistics” (5, 14.7%). Among company representatives, 32 answered this question with 28 comments. Most comments about issues to improve were related to “Meeting Organization and Structure” (9, 32.1%) and “Preparation and Logistics” (9, 32.1%). Figure 4 shows Q12 results by stakeholder group, and Table 4 highlights more representative comments.

**Table 4:** Comments to Q12: Should we have done any part of the meeting differently?

Category	CAB	Company representatives
Positive Comments	“No (need to change anything).”	“I appreciate the opportunity to listen and learn.”
Meeting Organization and Structure	“Not all points on the agenda were addressed.” “The order of the agenda was changed.”	“Ensure that all CAB participants have time to speak up and share their thoughts.”
Meeting Format and AV	“(We need) crystal clear audio and video with a bulletproof internet connection.”	“The audio with the hybrid approach can be difficult.”
Length of Meeting	“We need more time to talk and discuss. The meeting went so fast!”	“We had a lot to cover, so maybe we could meet more often in shorter increments, or longer meetings.”
Preparation and Logistics	“In the future, it would be better to receive pre-readings earlier.”	“More attention to meeting preparation and logistics” “Improve communication on the agenda.”

Abbreviations: AV, audio-visual; CAB, Community Advisory Board; Q, question

## Discussion

Overall, the feedback was highly positive. Most participants found the meetings useful, were satisfied with the topics addressed, and felt trust, transparency, openness, and a give-and-take relationship. CAB members exhibited slightly less favorable responses to all closed-ended questions vs. the company representatives. CAB members expected more from the meetings than they received compared to the company representatives. Likewise, regarding the possible influence of the meetings on companies’ actions, the CAB members’ opinions were less favorable than those of the companies. Both groups considered that the company understood the CAB’s point of view, but only 59.3% of CAB members believed the company would reconsider plans based on CAB input.

The lower confidence of CAB members on the possible influence of meetings on companies’ actions could, in part, be explained by prior experiences where CAB members’ priorities did not align with those of the companies. The previous 2022 study about EuroCABs’ Patient Engagement Monitoring and Evaluation Framework highlighted this difference [19]. While all parties agreed on the top priority—“Ensuring that products better meet unmet medical needs”—none of the other priorities fully aligned. For patients, the second and third priorities were “Access inequalities” and “Improve speed and efficiency of R&D”. In contrast, companies focused on “Enhancing transparency and trust” and “Making studies more patient-centered and reducing the burden of trials”. This might have made it more difficult to reach a clear agreement on the next steps, i.e., the actual impact on companies’ short-term plans. While CAB members’ concerns focused on their specific objectives, companies’ priorities encompassed broader, longer-reaching issues. CAB members expected a quicker turnaround on input/output; however, companies generally need more time to respond to CAB members’ suggestions. Companies also did not clearly express how the CAB input might be addressed internally over the longer timescale of the company. This could be a frustration for the CAB members and lower their satisfaction with the meetings.

The results for the open-ended questions reflected greater alignment between CAB members and companies. According to the comments to the question “What were your three key takeaways from this CAB meeting?” (Q11), CAB members and company representatives agreed on the three main takeaway areas, “Collaboration and Partnerships”, “Study Design and Protocols”, and “Communication and Education”, in line with previous studies pointing out the “Research Design” and the “Impact on the participants” (new skills and knowledge) as main effects of PE on R&D [39]. Besides, there was also reciprocity in both groups’ comments (see Table 3). Most participants highlighted the positive atmosphere and companies’ willingness to continue collaborating, as well as the opportunity to address specific trial-related issues. They also emphasized the need to promote education for patients and healthcare professionals, to globalize access, and to address mental health adequately. These results reflect agreement and collaboration, supporting the potential influence of CAB meetings on company actions.

These results, reflecting agreement and collaboration, are consistent with previous studies evaluating other PE initiatives, which have highlighted their positive effects on health products R&D. The EMA’s PE initiatives have demonstrated the importance of patient involvement in R&D. In a 2022 case study, they showed that patients contributed to selecting the clinical trial population, endpoints, study feasibility, and quality of life. Also, 20% of patients’ inputs modified the development plan [5]. Likewise, according to the results of the 24 specific-disease meetings conducted by the FDA’s PFDD initiative from 2012 to 2017, the FDA highlighted the benefits of incorporating patient perspectives into the drug development process and the resulting meaningful treatment benefits. They also recognized that these FDA-led meetings could not address the gaps in patient perspectives [40], which supports the need for more patient-led initiatives.

Finally, the issues identified in the question “Should we have done any part of the meeting differently?” (Q12, Table 4) highlighted that most participants agreed that not all scheduled topics could be covered in the allotted time, nor could all attendees fully participate. They also pointed out the logistical challenges of hybrid meetings spanning multiple continents, including poor connection and sound quality. They suggested holding longer or more frequent meetings and sending documentation on the agenda and pre-readings in advance. We believe that implementing these suggestions could ensure that all participants have more time to speak and fully address all the agenda points. This could help improve participants’ satisfaction with the meetings.

## Strengths and limitations

### Strengths

The seven-minute online survey format, the reminder email, and EUPATI Spain’s drafting of the questions based on the outcomes of the PARADIGM project [29] would have facilitated participation and minimized the number of non-responders. This allowed us to achieve an overall response rate of 54.8%, above the average for online surveys [41–43]. It has been shown that how questions are worded and the length of the questionnaire may affect their interpretation or response rate [31, 44–46]. Patient involvement in questionnaire design may reduce survey bias and lead to the co-design of more patient-relevant questions, thereby significantly impacting patient participation [44].

CAB members’ roles as patients or patient advocates supported the sample’s representativeness of the study population. This, together with the hybrid modality—which enabled participation from various locations—and the inclusion of both rare and non-rare diseases, broadened the scope of patients’ opinions and better reflected patients’ actual perspectives in the survey responses. In addition, anonymity allowed participants to answer more honestly.

Finally, the survey design, with combined closed-ended questions and open-ended questions processed by AI, allowed respondents to express themselves freely while facilitating quantification of responses and data comparisons.

### Limitations

The study’s main limitations were related to its design. Non-probability consecutive sampling could increase the total number of respondents, although it could skew the distribution across different CABs, with some over- or under-represented. In that case, some CAB-specific factors, such as disease characteristics, CAB development level, and goals, could influence the results. No subgroup analysis was conducted to study the potential effect of these factors. Although an analysis of this type could provide a more complete vision, it was beyond the scope of this study, which focused on assessing the differences between the CAB members group and the company representatives group. Therefore, the survey here collected data only on the stakeholder group, not on the disease-specific CAB. In addition, these results represent only a limited number of CABs and companies, and studies with more CABs and companies are needed to generalize them.

## Implications for the future

We will evaluate all comments from participants to improve the meetings and the CAB members’ confidence in the potential influence of CAB meetings on companies’ actions. Each CAB freely decides the content and format of the meetings and will assess whether to hold longer or more frequent sessions, depending on its specific needs, so that all attendees can participate and no issues are left unaddressed. In addition, the submission of pre-reading documentation and a list of questions to be discussed in advance will be encouraged. Regarding the challenges of hybrid meetings, the minute-taker and the recording capture the core points to share with all the participants. Beyond the co-design of programs and studies during the meetings, many ideas that are discussed in the meetings but cannot be implemented immediately will not be lost.

These results can be used for future studies, even in other disease areas. For other CABs and CABs in development, these data will be very useful for directing or redirecting guidance on meeting preparation, execution, and follow-up, as well as deciding how to evaluate the potential influence of CAB meetings on companies’ plans.

We will now address the next phase of questions to assess the actual impact of CAB meetings on the therapeutic development program, looking at questions such as:

- Do CABs shorten therapeutic development time?
- Do CABs change the question(s) asked in a development program?
- Do CABs offer a new way forward for approval?
- Do CABs help with access to new (approved) medicinal products?

With this goal in mind, we have developed a ‘Success Tracker’ tool that allows each CAB to monitor its specific objectives (see Additional File 5). Some CABs have already reported successes of CAB meetings (see Supplementary Table 2, Additional File 6).

## Conclusions

Overall, the satisfaction level and perceived usefulness of the CAB meetings were highly favorable. CAB members exhibited slightly less favorable responses to the closed-ended questions than the company representatives, especially regarding the possible influence of CAB meetings on future company actions. This lower level of satisfaction among CAB members may be due to prior experiences where priorities diverged, making rapid joint decisions difficult. CAB members may expect a quicker response from companies, which often need more time to fulfill their commitments. In addition, companies may not clearly report how they do/do not incorporate suggestions either in the short- and/or longer-term. In the open-ended questions, CAB members and company representatives agreed on the same three main takeaways (“Collaboration and Partnerships”, “Study Design and Protocols”, and “Communication and Education”), and there was reciprocity between both groups’ comments. These comments reflect a level of agreement and collaboration that supports the CAB model’s potential. We believe that disseminating these study results could increase CABs’ and companies’ confidence in the potential influence of CAB meetings on companies’ activities.

Finally, some issues, mainly related to the organization of the meetings and the hybrid meeting format, could have reduced attendees’ satisfaction and expectations, but they can be improved. We will evaluate and implement the participants’ suggestions to improve the meetings going forward.

## Electronic supplementary material

Below is the link to the electronic supplementary material.

Supplementary material 1

Supplementary material 2

Supplementary material 3

Supplementary material 4

Supplementary material 5

Supplementary material 6

## Data Availability

All data generated or analyzed during this study that are not included in this published article and its supplementary information files can be made available from the corresponding author upon reasonable request.

## Abbreviations

aHUS: atypical Hemolytic Uremic Syndrome
AI: Artificial Intelligence
AV: Audio-visual
CAB: Community Advisory Board
CF: Cystic Fibrosis
CFE: Cystic Fibrosis Europe
Company: Health product developers
COPD: Chronic Obstructive Pulmonary Disease
Dravet: Dravet Syndrome
EMA: European Medicines Agency
EUPATI Spain: European Patients’ Academy on Therapeutic Innovation Spain
EURORDIS: European Organization for Rare Diseases
FDA: Food and Drug Administration
LGMD: Limb-Girdle Muscular Dystrophy
MS: Multiple Sclerosis
PARADIGM: Patients Active in Research And Dialogue for an Improved Generation of Medicines
PE: Patient Engagement
PFDD: Patient-Focused Drug Development
Pulmonology: A cross-section of pulmonary diseases, including asthma, bronchiectasis, chronic obstructive pulmonary disease, and cystic fibrosis
Q: Question
R&D: Research and Development
