# Supplementary material for: Evaluation of the methodology of independent Community Advisory Boards in health products research and development: a mixed-methods cross-sectional survey study
Source: Res Involv Engagem. 2026 Mar 20;12:54. doi: 10.1186/s40900-026-00866-9 (PMC13126865; doi:10.1186/s40900-026-00866-9)
Supplement: Supplementary file 1 — Supplementary material 1 [file 40900_2026_866_MOESM1_ESM.pdf]

XX Community Advisory Board

Role of a CAB member and how to apply to join

Introduction

The XX Partnership is working with EUPATI Spain to set up an XX Community Advisory Board (CAB). This is an important initiative for XX for people affected both in Europe and globally. (EURORDIS started this as a European concept but many rare diseases are of global networks, so at EUPATI Spain we will expand). The XX CAB offers the following opportunities:

- To influence the research and development pipeline for XX treatments so that it better meets the needs of patients and carers.
- To increase access to, and reimbursement of, XX treatments across the globe.
- To improve awareness and understanding of XX, both across Europe and globally, within the healthcare system, academia, industry and EU institutions, and among the general public.
- To create a louder voice for the XX patient community.
- To recruit, train and support more patient advocates and experts to work within individual European countries and across the globe as a whole.

The XX CAB will be a group of patient advocates and expert patients/carers who use their personal and/or professional knowledge and expertise to discuss and advise on the latest developments, challenges and issues related to medical treatments and procedures under development in their disease area. The CAB will act as a consulting service to stakeholders involved in the research, development, reimbursement and service provision of biomedical treatments or processes, including both scientific and policy-related issues. The CAB has an important role in setting the research agenda for the future, based on patient needs.

EUROCAB Objectives

The objectives of the EuroCAB programme are to set up a high-quality standard for the interaction between sponsors of clinical research, public or private, and representatives of the patients. This will ensure that these interactions will create a high-quality dialogue between all parties, in the interest of the value of the research, and ultimately benefiting patients.

With this programme and these objectives, everyone, including policy makers and regulators, can be comfortable with the idea that sponsors and patients can work together on agreed

research objectives, as it enables a structured, opened, inclusive, transparent, patient-driven dialogue, based on mutual understanding and trust.

The EuroCAB programme provides

- Independence and efficiency – following the EuroCAB programme helps ensure independence, reliability and efficiency in the interaction with sponsors
- Transparency and commitment – the EuroCAB programme ensures that all patients know who represents them in clinical research discussions, who to contact if they need to do so, and that those who represent them follow up the research over time, from design to communication of results and through pharmacovigilance
- Support of policies and research centres – the EuroCAB Programme will establish a high-quality dialogue in the interest of patients
- Interoperability – the ability of Community Advisory Boards to operate and share experience relies on guidance and services provided by the EuroCAB programme
- Advocacy benefits – standardization provides a solid foundation upon which to develop patient engagement and gives consistent meaning to the term

This document sets out what is involved with being a member of the Community Advisory Board and related requirements.

Role description

Overview of role

CAB members act as a systematic, efficient, professional and accountable group of collaborators who speak with external stakeholders involved in research and development of lymphoma medical treatments and procedures, including with those involved in the design and running of clinical trials, as well as national and any institution globally dealing with reimbursement and access issues. CAB members, both individually and collectively, provide expert advice and insight in a neutral, objective and critically constructive manner, representing the unique perspective of the patient voice.

Main responsibilities or tasks

- To attend meetings of the CAB as and when required, both in person and by way of e-meetings or conference calls.
- To be an active participant in CAB meetings, sharing expert insight and advice with the external stakeholders attending the meetings.

- To commit to ongoing learning, training and development outside of CAB meetings so as to be part of a high quality, well-informed, influential and insightful CAB.
- To represent the interests of participants in clinical trials, including:
    - Reviewing clinical trial design at a planning stage.
    - Monitoring selected on-going trials with regard to patient priorities.
    - Staying informed about the interim results of selected trials, when appropriate.
    - Suggesting and helping start trials that reflect patient needs, and quality of life (QoL) and patient-reported outcome (PRO) measures.
    - Reviewing informed consent forms (in relation to appropriate content, language, logic, etc).
- To promote best practice in research procedures and ethics.
- To represent the patient perspective at investigator and advisory meetings and DSMCs.
- To advocate for fair, sustainable, and affordable pricing and access to treatment.
- To promote universal access to treatment, including for vulnerable groups (eg, children, the elderly, disabled people).
- To support the involvement of patients in adverse events reporting.
- To maintain strict confidentiality in relation to the CAB's work and any information, market insight or personal information shared with the CAB, unless otherwise cleared by the Chairs or Scientific leads of the CAB. Ie, always ask the Chair before sharing information outside the CAB.

Reimbursement/remuneration

CAB members will be paid for their time in attending formal CAB meetings at a day rate of €XX per day (or part thereof). Time spent travelling will not be reimbursed, however reasonable travel and accommodation costs will be reimbursed.

Key experience, knowledge, abilities and skills

- Excellent knowledge of XX gained from personal or professional experience.
- Good communication and personal interaction skills.
- Good advocacy skills.
- Ability to understand complex science, clinical trials processes and research/development issues.
- Ability to think, communicate and act objectively and constructively.
- Respect for others and integrity.
- A passion for improving outcomes in XX.

Eligibility to become a CAB member

1 To be a XX CAB member, you must

i) have, or have previously been diagnosed with XX; or
ii) be a parent of someone living with XX, or
iii) be a carer of a person with XX, or
iv) be a family member of a person with XX, or
v) be a member of an organisation working primarily in the field of XX (see also para 2 below).

a) be able to demonstrate a strong interest in the wider XX community and/or XX treatment research and development, eg, through being linked with, affiliated to or a member of an organisation working primarily in the field of XX, or through being part of established online social networks related to XX.
b) have good English skills, including reading, writing and conversing/interacting in the language.
c) be able to demonstrate an interest in, or willingness to learn about, science and medicine in order to fully appreciate the depth of the CAB's work. An interest in social sciences can also be important. (For those with limited scientific or medical knowledge/understanding, training and support will be provided, including through face-to-face and virtual training, and through access past presentations and online/distance learning provided through EURORDIS, EUPATI Spain and the Global XX Partnership.)
d) have the available time to commit to the work of the CAB, including any required training. The time commitment varies from week to week, and month to month, but on average CAB members should allow up to eight – ten hours per month.
e) have a willingness to travel to CAB meetings, which can be held in different places and can last for two, three or four days.
f) have a good internet connection to enable e-communication and e-meetings of the CAB.

2 If you are applying while a member of an organisation working primarily in the field of XX, please ensure:

a) You have the agreement of the authorised person within your organisation to apply and be part of the CAB, including making the necessary time commitment. You will act as an individual, but the backing of your local/national organisation is useful.
b) You will not be able to share any confidential information with anyone outside the CAB.
c) Your organisation adheres to the Code of Practice Guiding the Relations between Patient Organisations and the Healthcare Industry (signed by organisations that are members of the Patients' and Consumers' Working Party at EMA) (see attached).

How to apply to become an XX CAB member

If you are interested in applying to become a CAB member, please send:

- a copy of your CV; and
- a supporting statement setting out your motivation to be a CAB member and how you fit the above criteria.
