# Supplementary material for: Evaluation of the methodology of independent Community Advisory Boards in health products research and development: a mixed-methods cross-sectional survey study
Source: Res Involv Engagem. 2026 Mar 20;12:54. doi: 10.1186/s40900-026-00866-9 (PMC13126865; doi:10.1186/s40900-026-00866-9)
Supplement: Supplementary file 2 — Supplementary material 2 [file 40900_2026_866_MOESM2_ESM.pdf]

Supplementary Table 1 Community Advisory Boards

CAB	Rare disease (Yes/No)	Foundation Year	No. members	Members Countries	Specific Objectives
Tuberous Sclerosis Complex (TSC)[a]	Y	2017	8	Italy, Portugal, Spain	• QoL.<br>• Clinical Trials.<br>• Future research.<br>• Access to medicines.
Limb Girdle Muscular Dystrophy (LGMD)[a]	Y	2018	8	Finland, France, Germany, India, Italy, Netherlands, Norway, South Africa, Spain, UK, US	• Patient outreach, criteria for participation, and retention.<br>• Education on research.<br>• Clinical studies and their design (reduce biopsies, add MRIs, reporting results).<br>• Informed consent forms and processes.<br>• Compassionate use programmes.<br>• Gene therapy controls.<br>• Standards of care.
Cystic Fibrosis (CF)	Y	2018	13	Belgium, Finland, Germany, Hungary, Israel, Italy, Latvia, Netherlands, Poland, Portugal, Spain, Turkey, UK, Ukraine	• Faster and sustainable access for all, including Eastern Europe.<br>• New class of medications.<br>• Side effects.<br>• People (thankfully) aging.<br>• RWD and PROMs.<br>• Patient needs and patient preferences.

CAB	Rare disease (Yes/No)	Foundation Year	No. members	Members Countries	Specific Objectives
Duchenne Muscular Dystrophy (DMD)[a]	Y	2018	12 + 3 alternates	Belgium, Canada, France, Germany, Israel, Italy, Netherlands, Romania, Spain, Sweden, Turkey, UK, US	• To intensify focus on the non-ambulant DMD population, considering their specific challenges.<br>• To reduce the burden by implementing home visits/infusions where feasible and safe.<br>• Use of digital endpoints, i.e., SV95C.<br>• Muscle and cardiac MRI when feasible.<br>• To prioritize TFTs over NSAA.<br>• Early access/compassionate use programs.<br>• Sibling protocols.<br>• To monitor and mitigate issues around gene therapy.<br>• Combination therapies.
Multiple Sclerosis (MS)	N	2020	11	Belgium, Croatia, France, Ireland, Italy, Netherlands, Portugal, Romania, Spain	• To provide an expert patient perspective to industry and academics in MS research and development.<br>• To identify common research priorities that represent the needs of patients and caregivers.<br>• To ensure the design and running of clinical trials with strong patient involvement from the start.<br>• To support increased access to and reimbursement of effective and affordable treatments.
Psoriasis	N	2020	10	Croatia, Greece, Italy, Netherlands, Portugal, Slovenia, Spain	• QoL.<br>• Clinical Trials.<br>• Future research.<br>• Access to medicines.

CAB	Rare disease (Yes/No)	Foundation Year	No. members	Members Countries	Specific Objectives
Cystinosis[a]	Y	2020	14	Belgium, France, Germany, Ireland, Mexico, Netherlands, Spain, UK, US	• Patient materials.<br>• Patient information.<br>• Packaging.<br>• Studies and therapies.<br>• PPI review for research calls.
atypical Hemolytic Uremic Syndrome (aHUS)	Y	2021	14	Australia, Brazil, Canada, Egypt, Germany, India, Poland, Singapore, Turkey, UK, US	• To influence the research and development pipeline for treatments to better meet the needs of patients and carers.<br>• To increase access to, and reimbursement of, treatments worldwide.<br>• To improve awareness and understanding of aHUS within the healthcare system, academia, industry, governmental institutions, and the general public worldwide.<br>• To create a louder voice for the patient community.<br>• To recruit, train, and support more patient advocates and experts to work within individual countries and internationally.
Dravet syndrome	Y	2021	6	Croatia, Germany, Italy, Netherlands, Poland, Serbia, Slovenia, Spain	• Clinical study design.<br>• Endpoints and how they are measured.<br>• Factors that are meaningful to patients when measuring health and social outcomes (QoL).<br>• Access across Europe.

CAB	Rare disease (Yes/No)	Foundation Year	No. members	Members Countries	Specific Objectives
					• Drug-drug interactions.<br>• Precision medicine.<br>• Treatment development for the adult population.
Myotonic Dystrophy (MD)	Y	2022	6	Netherlands, Switzerland, UK, US	• To make studies more patient-friendly.<br>• Future research.
Hereditary Hemorrhagic Telangiectasia (HHT)	Y	2022	7	Germany, Ireland, Italy, Netherlands, Norway, Spain, Switzerland	• To better understand the symptoms.<br>• To find treatment for the disease.<br>• To avoid fragmented research.<br>• To avoid repetitions of research.<br>• To repurpose already approved medicines.<br>• To review scientific papers.
Pulmonology	Y/N	2023	10	Germany, Israel, Italy, Netherlands, Spain, UK, US	• Validity of studies.<br>• Clinical Trials.<br>• Future research.<br>• Access to medicines.

Abbreviations: MRI, magnetic resonance imaging; NSAA, North Star Ambulatory Assessment; PPI, Patient Public Involvement; PROM, patient-reported outcome measures; Pulmonology, a cross-section of pulmonary diseases including asthma, bronchiectasis, chronic obstructive pulmonary disease (COPD), and cystic fibrosis (CF); QoL, quality of life; RWD, Real World Data; SV95C, stride velocity 95th percentile; TFT, Timed-function tests.

[a] Autonomous CAB.
