# Supplementary material for: Evaluation of the methodology of independent Community Advisory Boards in health products research and development: a mixed-methods cross-sectional survey study
Source: Res Involv Engagem. 2026 Mar 20;12:54. doi: 10.1186/s40900-026-00866-9 (PMC13126865; doi:10.1186/s40900-026-00866-9)
Supplement: Supplementary file 3 — Supplementary material 3 [file 40900_2026_866_MOESM3_ESM.pdf]

GRIPP2 short form

Section and topic	Item	Reported on page No.
1: Aim	Report the aim of PPI in the study	Pages 5-6
2: Methods	Provide a clear description of the methods used for PPI in the study	Pages 6-10
3: Study results	Outcomes—Report the results of PPI in the study, including both positive and negative outcomes	Pages 12-15
4: Discussion and conclusions	Outcomes—Comment on the extent to which PPI influenced the study overall. Describe positive and negative effects	Pages 15-20
5: Reflections/ critical perspective	Comment critically on the study, reflecting on the things that went well and those that did not, so others can learn from this experience	Pages 15-20

PPI, patient and public involvement.
