# Supplementary material for: Evaluation of the methodology of independent Community Advisory Boards in health products research and development: a mixed-methods cross-sectional survey study
Source: Res Involv Engagem. 2026 Mar 20;12:54. doi: 10.1186/s40900-026-00866-9 (PMC13126865; doi:10.1186/s40900-026-00866-9)
Supplement: Supplementary file 4 — Supplementary material 4 [file 40900_2026_866_MOESM4_ESM.pdf]

# [redacted] EuroCAB / [redacted] meeting

Thank you for participating in our recent meeting.

We want to hear your feedback so we can keep improving. Please fill this survey by 10 Dec 2022 (your answers will be anonymous).

* Indica que la pregunta es obligatoria

1. Was the meeting with [redacted] on 21 November 2022 useful [redacted]? *

   *Marca solo un óvalo.*

   | | 1 | 2 | 3 | 4 | 5 | |
   |---|---|---|---|---|---|---|
   | Not | ◯ | ◯ | ◯ | ◯ | ◯ | Very much |

2. Were all the topics and points of discussion addressed to your satisfaction? *

   *Marca solo un óvalo.*

   | | 1 | 2 | 3 | 4 | 5 | |
   |---|---|---|---|---|---|---|
   | Not | ◯ | ◯ | ◯ | ◯ | ◯ | Very much |

3. Was the CAB well-prepared for the meeting? *

   *Marca solo un óvalo.*

   | | 1 | 2 | 3 | 4 | 5 | |
   |---|---|---|---|---|---|---|
   | Not | ◯ | ◯ | ◯ | ◯ | ◯ | Very much |

4. Was [redacted] well-prepared? *

*Marca solo un óvalo.*

| | 1 | 2 | 3 | 4 | 5 | |
|---|---|---|---|---|---|---|
| Not | ◯ | ◯ | ◯ | ◯ | ◯ | Very much |

5. How much did you feel trust, transparency and openness, shared learning and a give-and-take relationship between [redacted] & the [redacted] EuroCAB? *

*Marca solo un óvalo.*

| | 1 | 2 | 3 | 4 | 5 | |
|---|---|---|---|---|---|---|
| Not | ◯ | ◯ | ◯ | ◯ | ◯ | Very much |

6. Were your expectations of the meetings met? *

*Marca solo un óvalo.*

| | 1 | 2 | 3 | 4 | 5 | |
|---|---|---|---|---|---|---|
| Not | ◯ | ◯ | ◯ | ◯ | ◯ | Very much |

7. Does [redacted] understand the CAB's points of view? *

*Marca solo un óvalo.*

| | 1 | 2 | 3 | 4 | 5 | |
|---|---|---|---|---|---|---|
| Not | ◯ | ◯ | ◯ | ◯ | ◯ | Very much |

8. Will [redacted] reconstruct their plans based on CAB input? *

*Marca solo un óvalo.*

| | 1 | 2 | 3 | 4 | 5 | |
|---|---|---|---|---|---|---|
| Not | ◯ | ◯ | ◯ | ◯ | ◯ | Very much |

9. Will [redacted] continue to commit to more engagement with the CAB based on this meeting? *

*Marca solo un óvalo.*

| | 1 | 2 | 3 | 4 | 5 | |
|---|---|---|---|---|---|---|
| Not | ◯ | ◯ | ◯ | ◯ | ◯ | Very much |

10. How much influence, if any, do you feel CAB input could have on the future research and actions of [redacted]? *

*Marca solo un óvalo.*

| | 1 | 2 | 3 | 4 | 5 | |
|---|---|---|---|---|---|---|
| Not | ◯ | ◯ | ◯ | ◯ | ◯ | Very much |

11. What were your three key take-aways from this event?

_______________________________________________

_______________________________________________

_______________________________________________

_______________________________________________

_______________________________________________

12. Should we have done any part of the meeting differently?

Este contenido no ha sido creado ni aprobado por Google.

Google Formularios
