# Supplementary material for: Evaluation of the methodology of independent Community Advisory Boards in health products research and development: a mixed-methods cross-sectional survey study
Source: Res Involv Engagem. 2026 Mar 20;12:54. doi: 10.1186/s40900-026-00866-9 (PMC13126865; doi:10.1186/s40900-026-00866-9)
Supplement: Supplementary file 5 — Supplementary material 5 [file 40900_2026_866_MOESM5_ESM.pdf]

Success Tracker

Our success tracker

Priority Area	Goal	Collaboration	Action to do	Milestones / updates	Goal reached
Access					
Research / Clinical Trials					
QoL / PROMs					
Patient Support / Communication					

For a longer and better life with Cystic Fibrosis
www.cf-europe.eu
EURORDIS RARE DISEASES EUROPE
