# Supplementary material for: Evaluation of the methodology of independent Community Advisory Boards in health products research and development: a mixed-methods cross-sectional survey study
Source: Res Involv Engagem. 2026 Mar 20;12:54. doi: 10.1186/s40900-026-00866-9 (PMC13126865; doi:10.1186/s40900-026-00866-9)
Supplement: Supplementary file 6 — Supplementary material 6 [file 40900_2026_866_MOESM6_ESM.pdf]

Supplementary Table 2. Successes of CAB meetings

CAB	Change advised and implemented
Duchenne Muscular Dystrophy (DMD)[a]	Fewer biopsies, fewer tests and questionnaires, shorter time on placebo, better randomization ratios, use of digital endpoints, and studies in the non-ambulant population.
Cystic Fibrosis (CF)	Easier-to-read informed consent forms (ICFs), use of video explainers, better packaging, more efficient inhalers, and use of real-world evidence (RWE).
Cystinosis[a]	Use of standard therapy until Day 0 of new studies.
atypical Hemolytic-Uremic Syndrome (aHUS)	Patient-facing materials, study flexibility, travel support, and a more complete adverse events reporting process.
Dravet syndrome	Clearer concomitant medication reporting.
Limb Girdle Muscular Dystrophy (LGMD)	Expanding the age range to enter the study.

Abbreviations: CAB, Community Advisory Board.

[a] Autonomous CAB.
